# A multilocus genetic risk score for obesity: Association with BMI and metabolic alterations in a cohort with severe obesity

**DOI:** 10.1097/MD.0000000000034597

**Published:** 2023-08-11

**Authors:** Sabine Julia Maria Sag, Stephanie Mueller, Stefan Wallner, Christina Strack, Ute Hubauer, Margareta Mohr, Judith Zeller, Thomas Loew, Michael Rehli, Julia Wimmer, Martina Erika Zimmermann, Lars Siegfried Maier, Marcus Fischer, Andrea Baessler

**Affiliations:** a Clinic for Internal Medicine 2, University Hospital Regensburg, Regensburg, Germany; b Institute of Clinical Chemistry and Laboratory Medicine, University Hospital Regensburg, Regensburg, Germany; c Department of Psychosomatics, University Hospital Regensburg, Regensburg, Germany; d Clinic for Internal Medicine 3, University Hospital Regensburg, Regensburg, Germany; e Deutsches Patent- und Markenamt, München, Germany; f Department of Genetic Epidemiology, University of Regensburg, Regensburg, Germany.

**Keywords:** genetic risk score, obesity, single nucleotide polymorphism, weight

## Abstract

Genome wide association studies have identified numerous single nucleotide polymorphisms (SNPs) associated with obesity, yet effect sizes of individual SNPs are small. Therefore, the aim of our study was to investigate whether a genetic risk score (GRS) comprising risk alleles of SNPs identified in the GIANT consortium meta-analyses shows association with body mass index (BMI) and other BMI related metabolic alterations in a cohort with an extreme phenotype. Genotyping of 93 SNPs was performed in 314 obese individuals (mean BMI 40.5 ± 7.8 kg/m², aged 45 ± 12 years), participating in a standardized weight reduction program, and in 74 lean controls (mean BMI 24.6 ± 3.3 kg/m², aged 41.7 ± 13.4 years). Allele numbers of all 93 SNPs were added to a GRS. Anthropometric parameters, parameters of glucose/insulin and lipid metabolism were assessed standardized after a 12 hours fast. GRS was significantly different between controls and obese individuals (unweighted GRS: 86.6 vs 89.0, *P* = .002; weighted GRS: 84.9 vs 88.3, *P* = .005). Furthermore, linear regression analysis showed significant associations of GRS with BMI (*P* < .0001), weight (*P* = .0005), waist circumference (*P* = .0039), fat mass (*P* < .0001) and epicardial fat thickness (*P* = .0032), yet with small effect sizes (*r*² < 0.06). In conclusion, in our study GRS could differentiate between extreme obese and lean individuals, and was associated with BMI and its related traits, yet with small effect sizes.

## 1. Introduction

Genome wide association studies (GWAS) have revolutionized obesity genetic research and revealed until now >900 single nucleotide polymorphisms (SNPs) associated with body mass index (BMI).^[1–4]^ However, most of these variants, which are based on the common-disease common-variant hypothesis, are still far from fully explaining the heritability of complex traits and it has been assumed that many more gene variants need to be detected in the future.^[5]^ Meanwhile, due to their large population based sample size, recent large-scale GWAS are even able to identify gene variants with small effect size and low effect allele frequency.^[6]^ Taking into account the rather small effect sizes of individual SNPs and the polygenic character of weight regulation in common obesity, genetic risk scores (GRS) comprising alleles of several gene variants may be more representative for detecting associations with BMI and related traits.^[7,8]^ Furthermore, in contrast to cross-sectional study designs, an extreme phenotype design is theoretically considered to be more powerful for discovering associations between loci and phenotypes, because extreme phenotype sampling enriches the presence of causal variants in a dataset of a certain size, thereby increasing the probability of discovering associations.^[9]^

Hence, the aim of our study was to analyze whether a GRS, comprising risk alleles of the most validated central adiposity related loci, identified by the third GIANT analysis in 2015,^[3]^ shows association with BMI and other BMI related metabolic alterations in a cohort with extreme phenotype.

## 2. Material and methods

### 2.1. Study population

Individuals were participants of the “*Obesity Weight Reduction and Remodeling Study*,” an ongoing prospective longitudinal study evaluating excessive body fat for its pathogenic potential in terms of cardiometabolic diseases and assessing the effects of a considerable weight reduction on interactions in system´s biology. Details of the weight loss programs have been published earlier.^[10–12]^ Briefly, patients are included if they are ≥ 18 years old, present with a BMI ≥ 30 kg/m^2^, a constant body weight in the last 3 months, and if they signed declaration of consent. Patients are excluded if they have 1 or more of the following: 10% reduction of body weight in the last 6 months, cancer, pregnancy, therapy with steroids or thyroid hormones, known heart disease, known type 1 or type 2 diabetes, known inflammatory bowel, rheumatoid, or systemic diseases, known chronic renal failure, known liver diseases, history of psychiatric illness, or addiction to drugs or alcohol. For comparison, healthy normal weight control subjects (BMI 20–25 kg/m²) of similar age and gender distribution are also studied. They are recruited by flyers, advertisements and friend referrals. The study was approved by the local Ethics Committee (decision number 05/001) and all participants gave their written and informed consent. Anthropometric examinations are conducted at baseline, after 3 months (reduced phenotyping protocol) and at the end of the program (after 1 year). In the present analysis only data at baseline before weight reduction were used.

### 2.2. Biochemical and anthropometric phenotyping

Routine blood parameters are immediately measured at the Institute of Clinical Chemistry and Laboratory Medicine at the University hospital of Regensburg. At once, plasma samples are sampled and stored at -70 °C for the more laborious enzyme-linked immunosorbent assays as well as DNA extraction.

### 2.3. DNA extraction and quantification of case and control samples

All DNA extractions from peripheral blood samples were performed using the Gentra Puregene Blood Kit (Qiagen) according to the manufacturer´s instructions.

### 2.4. SNP genotyping of case and control samples

95 SNPs corresponding to loci identified in the first 3 GIANT meta-analyses of adult BMI^[1–3]^ were genotyped. For rs4836133 near ZNF608 and rs2867125 near TMEM 18 proxy SNPs were used, namely the proxy SNP rs939583 for TMEM 18 and the proxy SNP rs6864049 for ZNF608. Genotyping was not successful for rs11191560 near NT5C2 and rs4740619 near C9orf93. SNPs identified in the first 2 GIANT meta-analyses^[1,2]^ were genotyped using TaqMan® SNP Genotyping assays. Novel identified SNPs of the third GIANT meta-analysis^[3]^ were genotyped using the MassARRAY system (Agena Bioscience) combining iPLEX primer extension chemistry and MALDI-TOF mass spectrometry.

### 2.5. Definition of metabolic syndrome (MetS) and healthy obese

MetS was diagnosed according to the NCEP Adult Treatment Panel III.^[13]^ It requires the presence of central obesity with waist circumference ≥102 cm in men and ≥88 cm in women, dyslipidemia with triglycerides ≥150 mg/dL, and HDL-cholesterol <40 mg/dL in men and <50 mg/dL in women. Hypertension and hyperglycemia were diagnosed for blood pressure ≥130/85 mm Hg and fasting plasma glucose ≥110 mg/dL. MetS was diagnosed when at least 3 out of 5 metabolic abnormalities were determined. Diagnosis of metabolically healthy obese (MHO) was based on the definition of Karelis et al^[14]^ and thus was made when besides a BMI ≥ 30 kg/m² 4 out of the following 5 criteria were met: triglycerides ≤150 mg/dL, total cholesterol ≤200 mg/dL, HDL-cholesterol ≥50 mg/dL and ≤: LDL-cholesterol ≤ 100mg/dL and HOMA ≤1.95.

### 2.6. Statistical analysis

Statistical analyses were performed using SPSS statistical software package (SPSS 23.0, IBM SPSS Statistics, Armonk, New York) and JMP (Version 11, SAS Institute Inc., Cary, NC). Categorical variables were compared using the likelihood ratio test. Continuous variables of baseline data are reported as the mean and its standard deviation. GRS was calculated on the basis of 93 SNPs. Each SNP was recoded as 0,1,2 according to the number of BMI increasing alleles. Allele numbers of all 93 SNPs were added to an unweighted GRS. For the weighted GRS each SNP was weighted by its relative effect size (ß coefficient) on BMI obtained from GIANT meta-analyses.^[1–3]^ We calculated the weighted GRS by using the following equation: weighted GRS = (ß_1 ×_ number of risk alleles of SNP_1_ +ß_2_ × number of risk alleles of SNP_2_ + ….+ ß_93 ×_ number of risk alleles SNP_93_). Linear regression analysis was used to evaluate the predictive value of both the unweighted and weighted GRS in relation to BMI and other metabolic traits.

## 3. Results

Genotyping and calculation of GRS according to 93 BMI related SNPs (see Table S1, http://links.lww.com/MD/J427, Supplemental Content, which shows a listing of the 93 investigated BMI related SNPs) was performed in 314 obese participants and in 74 controls.

Baseline clinical characteristics are displayed in Table 1. Anthropometric parameters, parameters of glucose and lipid metabolism, adipocytokines and epicardial fat thickness were unfavorably altered in obese individuals compared to controls.

**Table 1:** Clinical and laboratory characteristics.

	Obese (n = 314)	Control (n = 74)
Age, yr	44.8 ± 11.9*	41.9 ± 13.4
Male number (%)	126 ± 40.1	28 ± 37.8
Weight, kg	119.2 ± 26.3**	71.7 ± 11.2
BMI, kg/m²	40.3 ± 7.5**	24.6 ± 3.3
Waist, cm	120.0 ± 18.9**	85.0 ± 10.2
WHR	0.9 ± 0.1**	0.8 ± 0.1
BP systolic, mm Hg	138 ± 18**	126 ± 15
BP diastolic, mm Hg	88 ± 12**	79 ± 10
Glucose, mg/dL	101 ± 30**	86 ± 9
Insulin, µU/mL	23.5 ± 21.3**	8.1 ± 5.3
HOMA	6.3 ± 7.4**	1.8 ± 1.3
Triglycerides, mg/dL	148 ± 85**	104 ± 64
Cholesterol, mg/dL	198 ± 36	204 ± 40
LDL, mg/dL	124 ± 32	117 ± 31
HDL, mg/dL	49 ± 14	64 ± 17**
Leptin, ng/mL	54.4 ± 50.0**	13.8 ± 13.5
Adiponectin, µg/mL	8.9 ± 4.0	11.5 ± 7.0**
Epicardial fat, mm	6.8 ± 3.2**	2.4 ± 2.2

Values represent the mean ± standard deviation or numbers (percentages).

BMI = body-mass index, BP = blood pressure, HDL = high density lipoprotein, HOMA = homeostasis model assessment, LDL = low density lipoprotein, WHR = waist to hip ratio.

* *P* < .05.

** *P* ≤ .001.

**Figure 1.**
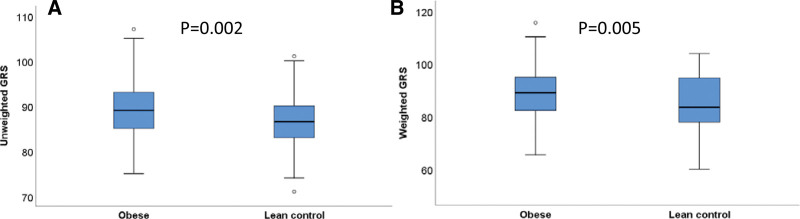
Box plots demonstrating unweighted (A) and weighted genetic risk scores (GRS) (B) in obese versus lean controls.

Combining all 93 genotyped SNPs into a GRS, we found a significant difference in the average score value for both unweighted and weighted GRS between control group and obese individuals (unweighted GRS: 86.6 vs 89.0, *P* = .002; weighted GRS: 84.9 vs 88.3, *P* = .005; Fig. 1A and B).

**Figure 2.**
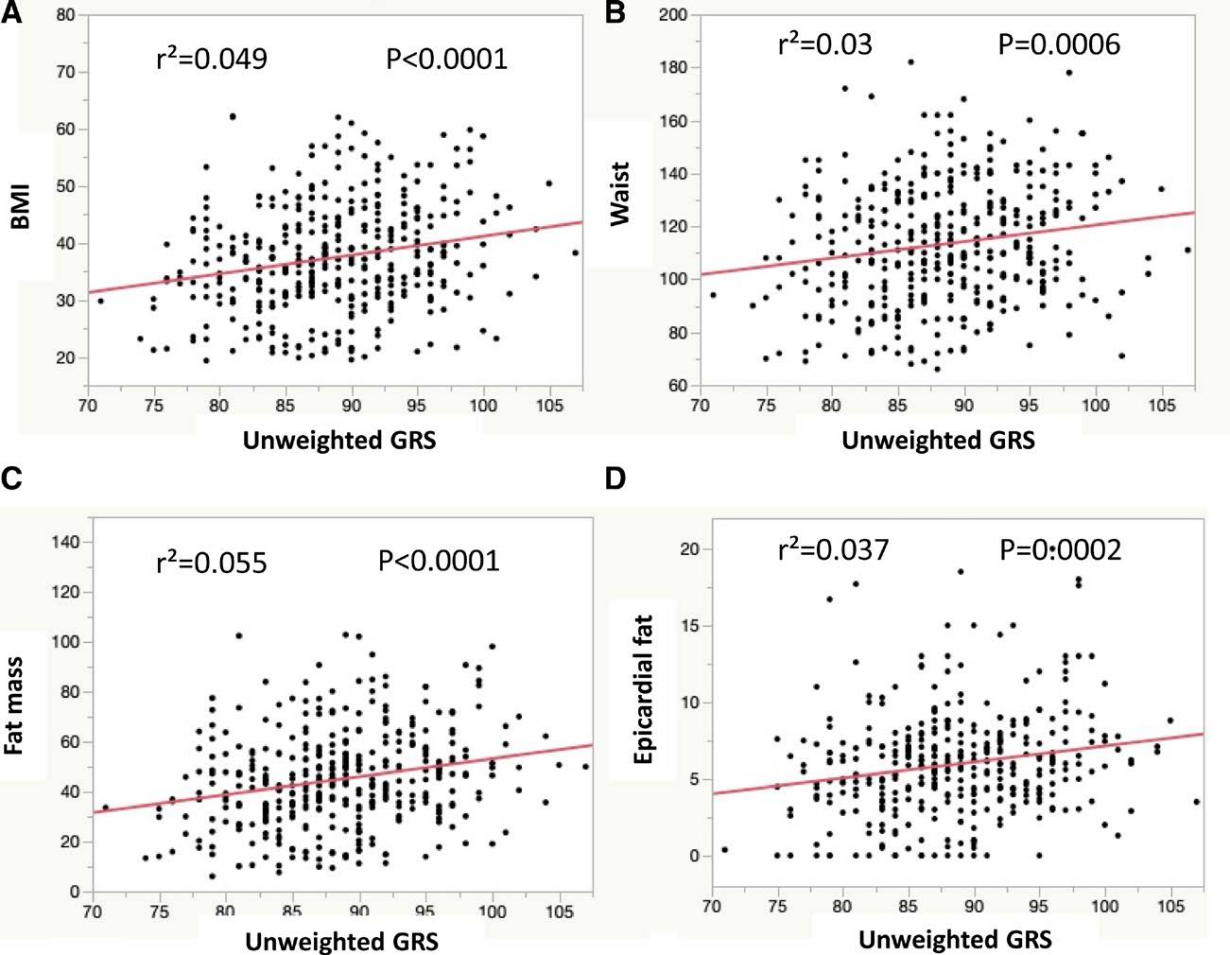
Scatter plots demonstrating relationship between unweighted GRS and BMI (A), waist circumference (B), fat mass (C), and epicardial fat thickness (D). BMI = body mass index, GRS= genetic risk score.

Linear regression analysis revealed for both unweighted and weighted GRS significant associations with BMI (both *P* < .0001) (Fig. 2A and Fig. S1b, http://links.lww.com/MD/J428, Supplemental Content, which shows relationship between weighted GRS and BMI), yet with low effect sizes (*r*² = 0.049 and *r*² = 0.04, respectively).

Unweighted and weighted GRS were also associated with BMI related traits like waist circumference (unweighted GRS: *P* = .0006 vs weighted GRS: *P* = .0039), fat mass (both *P* < .0001) and epicardial fat thickness (unweighted GRS: *P* = .0002 vs weighted GRS: *P* = .0032; Fig. 2B–D and Figure S1c-e, http://links.lww.com/MD/J428, Supplemental Content, which shows relationship between weighted GRS and waist circumference, weighted GRS and fat mass as well as weighted GRS and epicardial fat thickness), but again with low *r*² values (<0.06).

GRS value did not differ between MHO and non MHO (unweighted GRS: 89.3 vs 89.0, *P* = .784; weighted GRS: 88.0 vs 88.3, *P* = .881) nor between individuals with and without MetS (unweighted GRS: 88.8 vs 89.3, *P* = .512; weighted GRS: 87.9 vs 88.7, *P* = .456; Fig. S2a-b, http://links.lww.com/MD/J429, Supplemental Content, which contains box plots demonstrating unweighted and weighted GRS in participants with MetS vs without MetS and MHO vs non MHO, respectively).

## 4. Discussion

In this study we investigated the cumulative effect of 93 obesity associated genetic variants on different obesity measures and we found that a GRS comprising all risk alleles of these SNPs was associated with BMI and other related traits in our cohort with extreme phenotype (mean BMI 40.5 kg/m²), but with low effect sizes as measured by *r*². Furthermore, despite the fact that obesity plays a key role in MetS, GRS failed to differentiate between MHO and non MHO as well as between individuals with and without MetS in our study.

As common obesity is a polygenic disorder, single gene variants are not representative for assessing the risk of developing obesity.^[7]^ In fact, BMI is affected by a large number of gene variants each contributing a small portion to the total genetic risk.^[15]^ Therefore, variants have been aggregated into GRS, counting for each risk allele often weighted by their effect size.^[15]^ Several studies used GRS based on the 32 SNPs, mainly reported in the first GIANT meta-analysis.^[16–18]^ Belsky et al and Goumidi et al could show significant associations of GRS with BMI and obesity in general population.^[16,17]^ In contrast to these population-based studies, Mägi et al studied the 32 SNPs comprising GRS in a bariatric cohort with extreme obesity (mean BMI 48.4 kg/m²).^[18]^ In line with our results, GRS could differentiate between obese and lean individuals in this study. However, in contrast to our analysis GRS showed no association to BMI within the bariatric cohort.^[18]^

Furthermore, 2 recent studies, both conducted in obese Iranian cohorts, were able to show associations of GRS with BMI^[19]^ and other cardiometabolic risk factors.^[20]^ However both studies used GRS based only on a limited number of obesity related SNPs.

As mentioned earlier, in our study, GRS could not distinguish between participants with and without MetS or between MHO and non MHO. This result contrasts with studies by Zhao et al and Yang et al.^[21,22]^ Zhao et al demonstrated that GRS was capable to show an association to MetS in a children population from China,^[21]^ whereas Yang et al found in a population-based study that GRS was associated with non MHO.^[22]^ However, in contrast to our study, these studies used a GRS comprising only a few obesity related SNPs.

Unlike the previous mentioned studies, our calculated GRS was based on 97 SNPs identified by the third GIANT analysis in 2015.^[3]^ Song et al and Brankvist et al used this 97 SNP based GRS in longitudinal studies in general population^[23,24]^ and they could find an association of GRS with BMI over the course of time. However, to the best of our knowledge our study is the first to investigate the GRS comprising SNPs of the third GIANT analysis not in general population, but in a cohort with extreme obesity.

Although GRS values in our analysis were significantly different between obese and lean individuals and linear regression showed significant associations with BMI and other obesity measures, effect sizes were very small (*r*² < 0.06). One possible explanation could be, that as we used genetic variants for common obesity in a cohort with extreme phenotype (mean BMI 40.5 kg/m²), effect size of associations might be larger, when including loci of extreme obesity that do not overlap with SNPs of common obesity.^[15]^

Several further limitations of our study should be mentioned. First is the small sample size of our cohort could have resulted in a lack of power. Moreover, in the meantime much more gene variants have been detected that we have not included in our analysis.^[4]^

In conclusion, despite small sample size, GRS based on SNPs identified in the third GIANT analysis could differentiate between extreme obese and lean individuals in our analysis. However, effect sizes of associations with BMI and other obesity measurements were small.

## Acknowledgments

We appreciate the invaluable contribution of all study participants. We gratefully acknowledge the excellence technical assistance of Josef Simon.

## Author contributions

**Conceptualization:** Sabine Julia Maria Sag, Christina Strack, Ute Hubauer, Margareta Mohr, Judith Zeller, Thomas Loew, Marcus Fischer, Andrea Baessler.

**Data curation:** Ute Hubauer.

**Formal analysis:** Sabine Julia Maria Sag, Marcus Fischer.

**Investigation:** Sabine Julia Maria Sag, Stephanie Mueller, Stefan Wallner, Christina Strack, Margareta Mohr, Judith Zeller, Michael Rehli, Julia Wimmer, Martina Erika Zimmermann, Andrea Baessler.

**Methodology:** Marcus Fischer.

**Supervision:** Lars Siegfried Maier, Marcus Fischer, Andrea Baessler.

**Validation:** Ute Hubauer.

**Writing – original draft:** Sabine Julia Maria Sag.

**Writing – review & editing:** Stephanie Mueller, Stefan Wallner, Christina Strack, Ute Hubauer, Margareta Mohr, Judith Zeller, Thomas Loew, Michael Rehli, Julia Wimmer, Martina Erika Zimmermann, Lars Siegfried Maier, Marcus Fischer, Andrea Baessler.

## Supplementary Material






