# Supplementary material for: A multilocus genetic risk score for obesity: Association with BMI and metabolic alterations in a cohort with severe obesity
Source: Medicine (Baltimore). 2023 Aug 11;102(32):e34597. doi: 10.1097/MD.0000000000034597 (PMC10419793; doi:10.1097/MD.0000000000034597)
Supplement: Supplementary file 1 [file medi-102-e34597-s001.pdf]

Supplemental Content

Sag SJM et al.

A multilocus genetic risk score for obesity: association with BMI and metabolic alterations in a cohort with severe obesity

Table S1 Listing of the 93 investigated BMI related SNPs

Nearest Gene	Full gene name	Chr	rsid	EA
**FTO**	alpha-ketoglutarate dependent dioxygenase	16	rs1558902	A
**NEGRI1**	neuronal growth regulator 1	1	rs2815752	A
**TMEM18**	transmembrane protein 18	2	proxy_rs939583	T
**MC4R**	melanocortin 4 receptor	18	rs571312	A
**BDNF**	brain derived neurotrophic factor	11	rs10767664	A
**QPCTL**	glutaminyl-peptide cyclotransferase like	19	rs2287019	C
**MTCH2**	mitochondrial carrier 2	11	rs3817334	T
**RBJ**	DnaJ heat shock protein family (Hsp40) member C27	2	rs713586	C
**GPRC5B**	G protein-coupled receptor class C group 5 member B	16	rs12444979	C
**SH2B1_ATP2A1**	SH2B adaptor protein 1	16	rs7359397	T
**LRRN6C_LINGO2**	leucine rich repeat and Ig domain containing 2	9	rs10968576	G
**MAP2K5**	mitogen-activated protein kinase kinase 5	15	rs2241423	G
**TNNI3K**	TNNI3 interacting kinase	1	rs1514175	A
**RPL27A_STK33**	ribosomal protein L27a	11	rs4929949	C
**PTBP2**	polypyrimidine tract binding protein 2	1	rs1555543	C
**LRP1B**	LDL receptor related protein 1B	2	rs2890652	C
**KCTD1**	potassium channel tetramerization domain containing 1	18	rs29941	G
**SEC16B**	SEC16 homolog B, endoplasmic reticulum export factor	1	rs543874	G
**GNPDA2**	glucosamine-6-phosphate deaminase 2	4	rs10938397	G
**TFAP2B**	transcription factor AP-2 beta	6	rs987237	G
**TMEM160_ZC3H4**	transmembrane protein 160	19	rs3810291	A
**FAIM2**	Fas apoptotic inhibitory molecule 2	12	rs7138803	A
**ETV5**	ETS variant 5	3	rs9816226	T

Nearest Gene	Full gene name	Chr	rsid	EA
**FLJ35779_POC5**	POC5 centriolar protein	5	rs2112347	T
**FANCL**	FA complementation group L	2	rs887912	T
**CADM2**	cell adhesion molecule 2	3	rs13078807	G
**NUDT3**	nudix hydrolase 3	6	rs206936	G
**ZNF608**	zinc finger protein 608	5	proxy_rs6864049	A
**NRXN3**	neurexin 3	14	rs10150332	C
**PRKD1**	protein kinase D1	14	rs11847697	C
**HNF4G**	hepatocyte nuclear factor 4 gamma	8	rs4735692	A
**MRPS33P4**	mitochondrial ribosomal protein S33 pseudogene 4	20	rs13041126	T
**ADCY9**	adenylate cyclase 9	16	rs2531995	T
**GNAT2_GNAI3**	G protein subunit alpha transducin 2	1	rs17024258	T
**HS6ST3**	heparan sulfate 6-O-sulfotransferase 3	13	rs7989336	A
**MTIF3**	mitochondrial translational initiation factor 3	13	rs4771122	G
**RPTOR**	regulatory associated protein of MTOR complex 1	17	rs7503807	A
**ZZZ3**	zinc finger ZZ-type containing 3	1	rs17381664	C
**SLC39A8**	solute carrier family 39 member 8	4	rs13107325	T
**RABEP1**	rabaptin, RAB GTPase binding effector protein 1	17	rs1000940	G
**STXBP6**	syntaxin binding protein 6	14	rs10132280	C
**LMX1B**	LIM homeobox transcription factor 1 beta	9	rs10733682	A
**CLIP1**	CAP-Gly domain containing linker protein 1	12	rs11057405	G
**KCNK3**	potassium two pore domain channel subfamily K member 3	2	rs11126666	A
**ELAVL4**	ELAV like RNA binding protein 4	1	rs11583200	C
**HIP1**	huntingtin interacting protein 1	7	rs1167827	G
**EHBP1**	EH domain binding protein 1	2	rs11688816	G
**HHIP**	hedgehog interacting protein	4	rs11727676	T
**CADM1**	cell adhesion molecule 1	11	rs12286929	G
PRKD1	*protein kinase D1*	14	rs12885454	T
**PARK2**	parkin RBR E3 ubiquitin protein ligase	6	rs13191362	A

Nearest Gene	Full gene name	Chr	rsid	EA
**IFNGR1**	interferon gamma receptor 1	6	rs13201877	G
**MIR548A2**	microRNA 548a-2	5	rs1441264	A
**FIGN**	fidgetin, microtubule severing factor	2	rs1460676	C
**UBE2E3**	ubiquitin conjugating enzyme E2 E3	2	rs1528435	T
**RASA2**	RAS p21 protein activator 2	3	rs16851483	T
**ZBTB10**	zinc finger and BTB domain containing 10	8	rs16907751	C
**NUP54**	nucleoporin 54	4	rs17001654	G
**HIF1AN**	hypoxia inducible factor 1 subunit alpha inhibitor	10	rs17094222	C
**CREB1**	cAMP responsive element binding protein 1	2	rs17203016	G
**GDF15**	growth differentiation factor 15	19	rs17724992	A
**TLR4**	toll like receptor 4	9	rs1928295	T
**TDRG1**	testis development related 1	6	rs2033529	G
**RALYL**	RALY RNA binding protein like	8	rs2033732	C
**CBLN1**	cerebellin 1 precursor	16	rs2080454	C
**HSD17B12**	hydroxysteroid 17-beta dehydrogenase 12	11	rs2176598	T
**PMS2L11**	PMS1 homolog 2, mismatch repair system component pseudogene 11	7	rs2245368	C
**FHIT**	fragile histidine triad	3	rs2365389	C
**SBK1**	SH3 domain binding kinase 1	16	rs2650492	A
**NAV1**	neuron navigator 1	1	rs2820292	C
**ETS2**	ETS proto-oncogene 2, transcription factor	21	rs2836754	C
**SCG3**	secretogranin III	15	rs3736485	A
**GBE1**	1,4-alpha-glucan branching enzyme 1	3	rs3849570	A
**MAPK3**	mitogen-activated protein kinase 3	16	rs4787491	G
**PLCD4**	phospholipase C delta 4	2	rs492400	C
**ASB4**	ankyrin repeat and SOCS box containing 4	7	rs6465468	T
**EPB41L4B**	erythrocyte membrane protein band 4.1 like 4B	9	rs6477694	C
**AGBL4**	ATP/GTP binding protein like 4	1	rs657452	A
**RARB**	retinoic acid receptor beta	3	rs6804842	G

Nearest Gene	Full gene name	Chr	rsid	EA
**LOC100287559_BBS4**	Bardet-Biedl syndrome 4	15	rs7164727	T
**LOC284260_RIT2**	Ras like without CAAX 2	18	rs7239883	G
**GRP**	gastrin releasing peptide	18	rs7243357	T
**NLRC3**	NLR family CARD domain containing 3	16	rs758747	T
**ERBB4**	erb-b2 receptor tyrosine kinase 4	2	rs7599312	G
**GRID1**	glutamate ionotropic receptor delta type subunit 1	10	rs7899106	G
**TCF7L2**	transcription factor 7 like 2	10	rs7903146	C
**LOC285762**	ncRNA	6	rs9374842	T
**FOXO3**	forkhead box O3	6	rs9400239	C
**MIR548X2**	microRNA 548X2	13	rs9540493	A
**CALCR**	calcitonin receptor	7	rs9641123	C
**TAL1**	TAL bHLH transcription factor 1, erythroid differentiation factor	1	rs977747	T
**SMG6**	SMG6, nonsense mediated mRNA decay factor	17	rs9914578	G
**KAT8**	lysine acetyltransferase 8	16	rs9925964	A

Chr, chromosome; EA, effect allele; rsid, reference SNP identity document; SNP, single nucleotide polymorphism
