# Supplementary material for: A multilocus genetic risk score for obesity: Association with BMI and metabolic alterations in a cohort with severe obesity
Source: Medicine (Baltimore). 2023 Aug 11;102(32):e34597. doi: 10.1097/MD.0000000000034597 (PMC10419793; doi:10.1097/MD.0000000000034597)
Supplement: Supplementary file 2 [file medi-102-e34597-s002.pdf]

Supplemental Content

Sag SJM et al.

A multilocus genetic risk score for obesity: association with BMI and metabolic alterations in a cohort with severe obesity

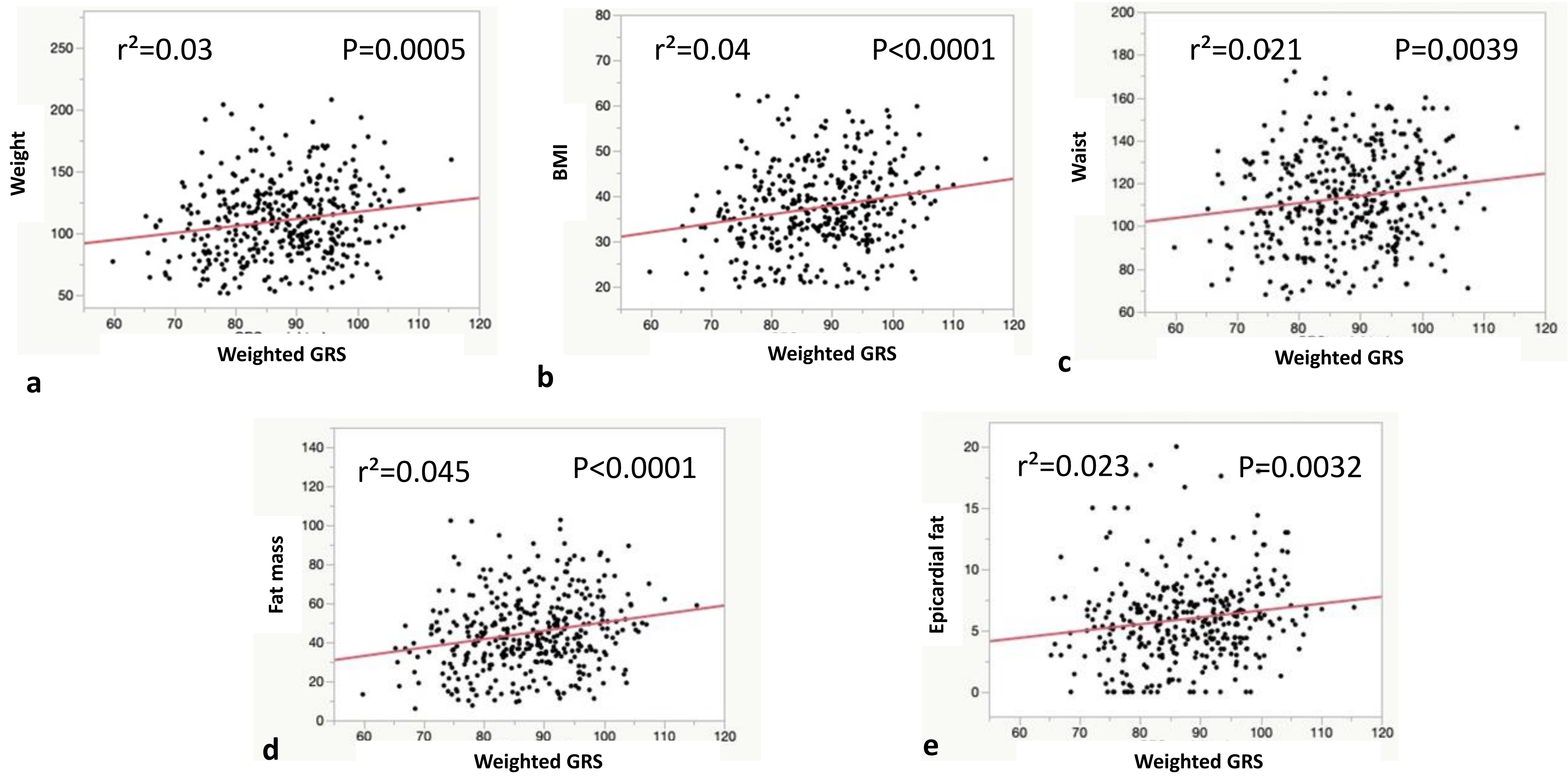

Figure S1 Scatter plots demonstrating relationship between weighted genetic risk score (GRS) and weight (**Figure S1a**), BMI (**Figure S1b**), waist circumference (**Figure S1c**), fat mass (**Figure S1d**) and epicardial fat thickness (**Figure S1e**)
