# Supplementary material for: A multilocus genetic risk score for obesity: Association with BMI and metabolic alterations in a cohort with severe obesity
Source: Medicine (Baltimore). 2023 Aug 11;102(32):e34597. doi: 10.1097/MD.0000000000034597 (PMC10419793; doi:10.1097/MD.0000000000034597)
Supplement: Supplementary file 3 [file medi-102-e34597-s003.pdf]

Supplemental Content

Sag SJM et al.

A multilocus genetic risk score for obesity: association with BMI and metabolic alterations in a cohort with severe obesity

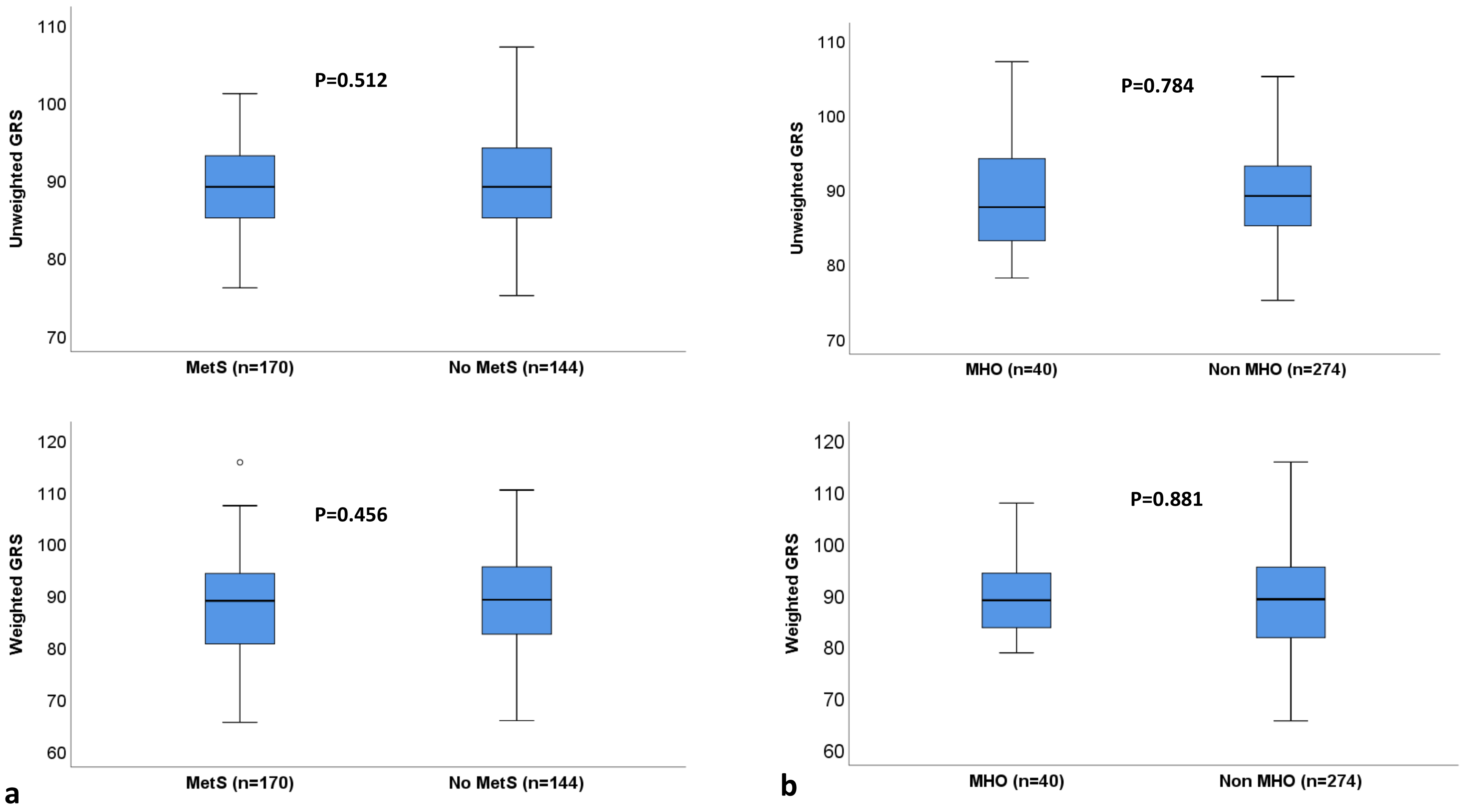

Figure S2 Box plots demonstrating unweighted and weighted genetic risk score (GRS) in participants with metabolic syndrome (MetS) vs. No MetS (**Figure S2a**) and metabolically healthy obese (MHO) vs. non MHO, respectively (**Figure S2b**).
